# Risk, governance and the experience of care

**DOI:** 10.1111/1467-9566.12017

**Published:** 2013-01-29

**Authors:** Alexandra Hillman, Win Tadd, Sian Calnan, Michael Calnan, Antony Bayer, Simon Read

**Affiliations:** 1 Cesagen, School of Social SciencesCardiff University; 2 School of Social Policy, Sociology and Social ResearchUniversity of Kent; 3 School of MedicineCardiff University

**Keywords:** clinical governance, risk, care, dignity, older people

## Abstract

Drawing on perspectives from the governmentality literature and the sociology of risk, this article explores the strategies, tools and mechanisms for managing risk in acute hospital trusts in the United Kingdom. The article uses qualitative material from an ethnographic study of four acute hospital trusts undertaken between 2008 and 2010 focusing on the provision of dignified care for older people. Extracts from ethnographic material show how the organisational mechanisms that seek to manage risk shape the ways in which staff interact with and care for patients. The article bridges the gap between the sociological analysis of policy priorities, management strategy and the organisational cultures of the NHS, and the everyday interactions of care provision. In bringing together this ethnographic material with sociological debates on the regulation of healthcare, the article highlights the specific ways in which forms of governance shape how staff care for their patients challenging the possibility of providing dignified care for older people.

## Introduction

This article highlights the relationship between the management of risk in healthcare institutions and the provision of dignified care to older people on acute hospital wards. Specifically, relations of care between staff and their patients are shown to be mediated by practices that seek to manage the uncertainties of clinical care. It is this mediation that poses a challenge to the possibility of maintaining dignified care for older people.

The risks posed by poor health outcomes are a major health policy priority across the UK and internationally (Allsop and Jones 2008). This is not least due to massive costs incurred for remedial care and compensation (Waring 2007). As a consequence, throughout the developed world, we have witnessed the design of systems that limit the capacity for individual discretion to manage risks to patient safety (McDonald *et al.* 2006).

In the UK these risk-management techniques have been politically accounted for on the basis that they engender confidence and secure public trust in care institutions (Checkland *et al.* 2004, Brown 2008a, Brown and Calnan 2009). The utility of such techniques as a means of improving health outcomes, maintaining quality or engendering trust has been challenged within social science research. This body of work is founded upon an analysis of health policy and governance strategies which challenges underlying assumptions and raises questions about: the nature of risk (Alaszewski 2006, Brown and Calnan 2009), the nature of trust (Smith 2001, Checkland *et al.* 2004, Alaszewski 2006, Brown 2008a) and the implications of the utilisation of these concepts in the context of health services; the increased rationalisation of healthcare provision (Germov 2005, Pinder *et al.* 2005, Brown 2008b); the alterations to professional practice, expertise and relations of care (Flynn 2002, McDonald *et al.* 2006, Waring 2007); the erosion of embodied, tacit knowledge and those more qualitative aspects of interpersonal communication (Ahmad and Harrison 2000, Pinder *et al.* 2005, Brown 2008a, Nettleton *et al.* 2008). This work provides an analytical critique of health service policy and governance strategies, complimented by some empirical studies highlighting the effects of these processes on professional practice (Germov 2005, Pinder *et al.* 2005, McDonald *et al.* 2006).

What is missing from this debate is ethnographic material that illustrates the relationship between mechanisms for healthcare regulation, clinical practice and experiences of care. This article highlights the conditions, environments and relations of care that governance strategies create and their consequences for the maintenance of patients’ dignity while being treated on acute wards.

### The sociology of risk, governmentality and the regulation of healthcare

Mechanisms for managing uncertainties have always formed part of healthcare practice (Fox 1959, Atkinson 1984, Paget 1988). What is significant about more recent healthcare provision is the shift that has occurred over how to manage this uncertainty. Health service policies over the past decade have attempted to eradicate uncertainties by building systems of regulation including the adoption of protocols, monitoring, targets and performance measures to achieve a more standardised practice (Checkland *et al.* 2004, Brown & Calnan, 2009). In the UK, these developments have, in part, been in response to a loss of confidence in medical professionals generated by a number of high profile scandals during the 1990s (see Checkland *et al.* 2004). The pressures on the NHS from increasing numbers of older people, chronic illness and technological advancement (Osborne 1993) meant that such scandals provided credence to the state’s pre-existing motivations for reordering clinical work. New Labour’s shift from government to governance in public services in particular (Walshe and Shortell 2004) drew upon these scandals to further their ‘modernisation’ agenda. Successive governments’ responses to these critical events have meant that internal strategies to regulate clinical risk have been brought into the arena of public scrutiny.

To understand the implications of such systems for the provision of dignified care, the article draws upon key sociological theories of risk. While recognising the diverse philosophical groundings of risk theorists, the article highlights possibilities for synthesis to show the utility of such ideas in understanding the relationship between risk regulation and patient dignity.

The particular understanding of risk that these regulatory systems are founded upon is amenable to control through instrumental, rational means (Beck 1992): a medical and, to an extent managerial, risk episteme that is assessed according to measures of probability embedded in a framework of instrumental reason. Weber’s theory of the rationalisation of society (Brubaker 1984) and Beck’s (1992) understanding of risk as an ontological condition of contemporary social forms together imply that all mechanisms for managing risk can be thought of as instances of instrumental rationality that may have de-humanising effects. The utility of Beck’s work in this context (building on Weber’s theory of rationalisation) is in recognising the impact of risks through the ways in which they are constructed by healthcare institutions, managers and practitioners in acute care. It is therefore the effects of rational systems of risk governance and their implications for care relationships that are distinguished in the ethnographic material presented.

Douglas (1992), like Beck, recognises the affects of risk as being born out through its construction by actors in social groups. For Douglas, risk and risk analysis have become embedded in all aspects of contemporary life: medicine, managerialism and the regulation of healthcare are good examples. It is a particular understanding of risk, based on a rational choice model of human behaviour, which Douglas argues denies the essential social determinant of risk and our human response to it. This conception of risk has shaped our relationship to blame so that every accident must hold within it a fault to which someone is held to account. This article highlights some of the de-humanising effects of such an understanding of risk and the constant presence of potential blame held within it.

In more recent years, theorists writing within ‘the governmentality tradition’ have developed analyses which can usefully be combined with Beck’s and Douglas’s work to examine the specific manifestations of risk-thinking within contemporary systems of governance. The governmentality of risk contends that power is exerted through actors’ negotiation of risk rationalities. This understanding shifts the focus away from risk itself onto the mechanisms, tools and techniques for managing risks (Peterson 1997, Dean 1999) and how these shape human conduct (Dean 1999). Like Douglas (1992), contributions from within governmentality literature explore the cultural shift in care institutions as a consequence of contemporary understandings of risk. For example, face to face relationships between the carer and the cared for – are compromised in favour of the collation of abstract factors deemed liable to produce risk (Castel 1991).

The ethnographic material drawn upon here highlight the nuances of everyday practices on acute wards where practitioners negotiate as well as execute governance agendas. Forms of management through self-surveillance have become a part of the culture of clinical practice (Waring 2007). It is therefore more useful to think about clinical governance as a form of governmentality in which the processes of self-audit and self-surveillance are themselves technologies of control (Rose 1997, Shore and Wright 1999). Thus, the codification and standardisation of clinical practice (Flynn 2002) is carried out with the ‘co-optation’ of medical professionals.

This article is concerned with the ways in which staff’s negotiations with systems of governance mediate their relationships with patients. It is important to acknowledge that the governance of risk exists among other, competing sets of rationalities that inform and influence staff actions. It is the tensions between these competing interests and the ways in which staff respond to them that shape staff-patient interactions and thus the patient’s experience of care. Hospital staff are immersed in what Horlick-Jones (2005a) has termed an ‘interactional matrix’ of moral duty, social obligation and institutional priorities and the matrix itself is shaped by the situations in which care is provided.

### Risk, governance and dignified care

Delivering on the promise of dignified care in the context of healthcare practice has often resulted in breaking down the meaning of dignity into component parts such as respect, autonomy, privacy and self-worth. In this article, dignity is understood to be maintained or challenged through social interaction. What dignified care actually comprises of in practice must therefore be viewed in context, taking into account the interactional situation, local cultures and the relationships between social actors. The article builds on the concept of ‘dignity of identity’ which Nordenfelt (: 75) 2003 describes as ‘the dignity we attach to ourselves as persons with a history and persons with a future’. Utilising this idea, dignified care is understood to be maintained through the maintenance of personal and social identity (Goffman 1963). Concepts such as autonomy or respect are therefore understood to be the emergent properties of interactional experiences rather than fixed moral codes. Such a conception of dignified care makes it possible to take account of local contexts and the broader institutional cultures in which care is delivered.

The relationship between staff behaviour and dignified care highlighted in nursing ethics literature (Baillie 2007, Gallagher *et al.* 2008) supports the idea of dignity as something maintained in social interaction. However, cultural and institutional contexts of healthcare delivery are often missing in these accounts and the maintenance of dignified care has, as a consequence, been focused upon individual attitudes and behaviours. This article highlights the cultural contexts in which staff deliver patient care on acute wards and illustrates how the possibilities for maintaining dignity depend on more than the commitment of individuals.

## The study context and methods

The ethnographic material analysed in this article is drawn from a study exploring dignity and the dignified care of older people in acute hospital wards in England and Wales (Tadd *et al.* 2011). The need for dignified care for older people has been recognised in recent health service policy (DoH 2006, 2010b,). However, the effectiveness of these policies in changing practice appears to be limited as recent evidence (CQC 2011, Health Service Ombudsman 2011) suggests that the NHS is failing to treat older people with care, dignity and respect.

Evidence from practitioners has identified the salience of professional, organisational and institutional influences on dignity in practice (see Tadd and Bayer 2006) but there is limited observational research of practices and little reflection on influences of the care environment on the provision of dignified care. This study pays attention to the contexts in which acute care is delivered and in doing so risk, and the regulation of risk, emerged as a central theme for understanding the everyday practices of delivering care and how older people experienced them.

The study received ethical approval from a NHS National Research Ethics Service (NRES) committee. This committee was responsible for evaluating the ethics of the entire project, taking account of all four NHS trusts involved. The project was also subject to governance procedures in each of the four trusts. The study took an ethnographic approach to reach the meaning and practice of dignified care as well as the political, organisational and cultural conditions in which they occur. Four NHS trusts in England and Wales participated, with four acute wards selected in each trust; 16 in total. The four sites were purposively selected to reflect a range of organisational and system characteristics which may impact on the provision of dignified care. These characteristics included: ratings of quality of care and resource use; organisational characteristics including acute trust status and the populations they served; as well as local trust initiatives directly related to dignified care for older people. The four trusts chosen had diverse characteristics both in terms of their formally rated performance ranging from ‘excellent’ to ‘weak’ and their local initiatives for delivering dignified care ranging from no activity at the time of the study to substantial organisational commitment. In each trust, the four wards selected in consultation with senior trust staff included two wards specifically for older people and two wards where all adults were treated.

Across the four trusts 40 older people, recently discharged from one of the 16 wards and 25 relatives/carers were interviewed. The 40 patients were comprised of an equal number of men and women with an average age of 74.5. Participants took part in the study voluntarily and were asked to provide an account of their time in hospital and to describe their experiences, both positive and negative. Similar themes were explored in interviews with relatives/carers who were also asked about their caring role and responsibilities.

Trust managers from all four trusts were interviewed and recruited on the basis of their hierarchical position and responsibility for patient experience. Table 1 outlines the roles of the 32 managers interviewed.

**Table 1:** The roles of the managers interviewed

Site 1	Site 2
Chair of the Board	Chief Executive
Director of Medicine	Chief Executive
Director of Nursing	Director of Medicine
Director of Finance and Information	Director of Nursing
Complaints/PALS Manager,	Director of Finance
Consultant Nurse for Older People	Patient Experience Manager
Matron	Director of Operations,
	Human Resources Manager
	Director of Service Planning
	Director of Pharmacy &; Facilities,
	Consultant Nurse for Vulnerable Adults
Site 3	Site 4
Chief Executive	Chair of the Board
Complaints/PALS Manager	Director of Nursing
Director of Nursing	Orthopaedic Matron
Matron	PALS/Volunteer Manager,
Director of facilities	Equalities Officer
	Associate Director of Learning and Occupational Development
	Surgical Matron
	Practice Development Matron
	Associate Director of Facilities and Performance

Managers discussed their individual role and responsibilities, the trust’s approach to ensuring quality and what they believed were the broader influences on dignified care provision for older people. Seventy-nine ward staff were interviewed across the 16 wards and their selection ensured a broad range of occupational groups (see Table 2).

**Table 2:** Job titles of hospital ward staff

	Site 1	Site 2	Site 3	Site 4
Ward Managers/deputies	4	4	4	3
Senior Nurses	1	2	0	0
Nurses	7	4	2	6
Student Nurses	3	0	2	1
Health Care Assistants	4	4	3	3
Domestic Staff	1	1	3	0
Occupational Therapist/OT Assistant	2	0	0	0
Physiotherapist/ Physio Assistant	0	4	1	0
Dietician	0	1	0	0
Activities Co-ordinator	1	0	0	0
Receptionists	3	0	0	0
Doctors	1	2	1	1
Total	27	22	16	14

Ward staff interviews explored understandings of dignified care, what detracts or enhances their ability to provide dignified care, staff training and support and their position within the organisation as a whole. These qualitative interviews were complemented by 617 hours observing practices and activities across the 16 wards (approximately 30-40 hours on each ward) covering the 24/7 period (see Table 3).

**Table 3:** Hours of observation

	Site 1	Site 2	Site 3	Site 4	Total
Hours of Observation	142.5	142	156.5	176	617

These unstructured (Bryman 2008) observations identified aspects of ward activity that shaped care delivery to older people, although certain aspects of care were identified as areas of interest by the literature and the previous research (Bayer *et al.* 2005). The field notes included observation of practices associated with enhancing, maintaining or detracting from the older person’s identity (e.g. being recognised and respected) and/or from their independence or autonomy.

The data were collected iteratively so that analysis informed the data collection and vice versa. Both field notes and interview transcripts were pooled from each of the four sites and analysed thematically aided by Nvivo-8. Researchers simultaneously analysed interview transcripts and observational field notes while carrying out fieldwork. This meant that emerging organisational issues, such as the regulation of clinical care and the management of risk, could be read and interpreted alongside everyday instances of acute care provision.

Researchers reflected upon how their own position as observer influenced the practices of care they observed and the impact their presence on the wards had on the dignity of both staff and patients. Intimate personal care provided to older people behind curtains or behind closed doors was never observed by researchers. When observing ward activities, researchers introduced themselves to patients and staff and explained the focus and purpose of the study. Researchers found that although some staff behaved in ways suggesting an awareness of the researcher’s presence initially (for example, referring to privacy and dignity policies while talking to patients or relatives), this awareness faded quickly. This was particularly the case during busy periods where the habits and routines of everyday practice took over.

Researchers were sensitive to the vulnerabilities of participants and withdrew when observation felt inappropriate (for example while family members were sitting with their dying relative). Decisions over when to observe, how to stay attuned to the wishes of those being observed and when to withdraw altogether were continually negotiated in the field between the researcher and the patient and staff participants. This approach to the process of obtaining and maintaining consent from research participants – that recognises consent as a process of continual negotiation – reflects the nature of qualitative research whereby not all potential ethical dilemmas can be anticipated at the outset (Renold *et al.* 2008). These ethical decisions, as well as the role researchers’ played in the care practices they observed, were recorded in the field notes and informed the interpretation of meaning in the data.

For the purposes of rigour, the data collected together within themes were checked for the consistency and validity of interpretation. The constant comparative method (Silverman 1993) was used to check the relationship between concepts and to build common themes across the four trusts. Initial analysis was presented to practitioners, managers and service user representatives during three workshops held in different locations across the UK. These workshops checked researchers’ interpretations and descriptions with participants as a means of ‘respondent validation’ (Bloor 1978). Alongside the internal methodological mechanisms for assessing study validity, respondent validation ensured that project themes resonated with the experiences of those in the field. Most of the findings, particularly the systemic factors that impinged upon staffs’ ability to provide dignified care, resonated with the workshop participants. There were some tensions and defensiveness among the frontline staff as observational examples were delivered. However, through open discussion between participants, many were able to recognise and articulate the problematic cultures of acute care in which they were entrenched.

The ward observations drawn upon in this article mainly involve healthcare assistants (HCAs) and nursing staff. Due to the nature of their work, these staff were situated on the wards full time unlike doctors and therapists who moved around the hospital. Our methodological approach was to situate researchers on the wards alongside patients in order to experience the ward environment from the patient’s perspective. Only on limited occasions did researchers follow members of staff in their movements outside the wards. The inevitable consequence of this was a focus on nursing staff and HCAs in our observations. This focus consequently shaped the interpretations of the data so that the relationship between risk, regulation and caring practices is viewed in the context of professional groups, hierarchies and local organisational practices on acute wards.

The following three sections present extracts from interview transcripts and field notes. Each section highlights a specific effect of technologies of risk and their negotiation in everyday practices of care. These effects are explored in the context of relations of care between staff and patients and the maintenance of patient dignity.

## Distorting practice

Performance measures and acute trust targets formed a central component of risk management through regulatory means in all four of the trusts involved in our study. There are, however, huge difficulties in measuring ‘quality’ in healthcare and choosing what to reward and value becomes a significant bureaucratic exercise. The immeasurability of the patient’s experience means it is inevitable that regulatory regimes are unable to reward or audit the things that matter (Brown and Calnan 2009), hence the inherent problems of relying on audit and measures to record and improve the patient experience. The data explored below illustrates how measurement can become a central risk technique, shaping the relationships between clinical staff and patients on acute wards in ways that can detract from dignified care.

To take an example: one of the key concerns of all four trusts was controlling hospital acquired infection. This resulted in many audits and measures to manage infection risk effectively. Every participating ward displayed graphs of their performance in key areas of patient safety, including rates of infection. This focus on infection prevention impacted on older people’s experiences of care resulting in increased isolation, low mood and confusion from being nursed in side-rooms or being moved around or between wards. Many patients interviewed or observed on the wards experienced such isolation and the limited contact with others was often exaggerated by restricted visiting, which was another response to reduce infection. Lilly explains her feelings of loneliness and isolation from being barrier nursed in a side room:

You wouldn’t want to be in a little room on my own because you felt lonely, on your own. You’re not feeling well. If there’s something going on around that you can watch it takes your mind off it. But that’s the only time I’ve cried in hospital being in a little room on my own. No I didn’t like it (Patient Interview: Site 1).

From the ward observations it was apparent how people in side rooms were often neglected and experienced less engagement with staff and other patients:

The side rooms are difficult to manage as they are rather isolated and tend to get left. Nobody seems to check the side rooms unless they call (Observation: Site 2).

Nurse Taylor, a ward sister, felt that patients’ needs came last due to a disproportionate focus within the Trust on infection control:

Nurse Taylor showed the researcher round the bays and pointed out that there were no lockers anymore as they had been told to remove them as part of the ‘de-cluttering’ drive. She said she tried to keep hold of them for as long as possible. It means now that patients have to keep everything on their table, which becomes very cluttered or in the cupboard in the wall which they cannot reach (Observation: Site 1).

Concentrating on measures to increase confidence has dysfunctional consequences. The most obvious of these is the potential for the distortion that occurs when particular performance targets, such as infection control, are privileged. This can result in those aspects of practice that are not or cannot be measured, becoming less visible. This invisibility is not only reflected at the level of trust management strategy, but is also in ward practices. The subtleties of recognising, respecting and treating older people with dignity, as the cases above describe, are obscured by target driven rates of infection.

The concerns over patient falls and other untoward incidents (also subject to performance measures) further distorted the provision of care on acute wards. This distortion manifested itself in a culture of restriction and limitation where patients were encouraged to remain in their chairs and use bedpans or commodes rather than be helped to a toilet. Staff were also more likely to use bed rails when perhaps they were not necessary:

I think that staff are very safety conscious and if bedrails are on beds, they think they need to pull them up and if they leave them down that could be being neglectful, even though that actually might be the right decision for that patient and there might still be risks, it’s about balancing them (Trust Manager Interview: Site 1).

This Trust manager describes a scenario evident throughout our observations. The concerns over patient safety, particularly for confused patients, meant that much staff time was spent preventing people from leaving their chairs in case they fell or interfered with equipment posing risks to others:

Phillip is standing up and is trying to walk. Both the SN (Staff Nurse) and the SSN (Senior Staff Nurse) rush to him and take him gently back to his chair, saying ‘There’s a cup of tea coming round in a minute’ (I later discover Phillip is a wanderer and has had a number of falls.) Phillip has got up again; he says he is going next door. The HCA says ‘I need you to sit down, will you sit down for me? Stay there for a bit, stay there for me’ (Observation: Site 3).

These constant attempts to control confused patients and get them to sit in one place sometimes lead to increased agitation (Marshall and Allan, 2006), inappropriate medication and reduced the possibility of a positive care experience for those patients.

The distortion of caring practices is shown to significantly alter the conditions in which staff and patients relate to one another and build relationships. The next section explores the disconnection that can occur between staff and patients when institutional risk regimes mediate the everyday practices of delivering care to older people.

## Disconnecting clinical relationships and de-humanising patients

Below are some examples taken from observations on the wards that illustrate how staff interpret and respond to the rationalities of institutional risk in their everyday practice, often detracting from their relationships with patients:

Annie calls out again and Amy (HCA) goes to her.

‘Can I go to the toilet please?’

‘You’ve got a pad on.’

‘Can I have help to the toilet please?’

‘If you … (she sighs with frustration) you’ve got low pressure, when you stand up your blood pressure drops and you’ll be falling’

(Observation: Site 2).

In this example, Annie’s risk of falling is prioritised over the indignity of being told to soil herself and the feelings of degradation that must naturally follow. Helping Annie and patients like her to the toilet was a timely and labour-intensive task. The official line for care delivery on acute wards was one of ‘twenty-four hour care’ where times for washing, dressing and eating were under the patient’s control. Paradoxically, the organisational structures of acute wards (and hospitals) meant that meals were delivered at set times and the distribution of those meals had to be completed within a specific time period, nurses had set times to complete their observations and the work of doctors and therapists was dependent on patient care ‘tasks’ being completed. As a result, speed in completing caring ‘tasks’ was highly valued. Discourses of risk and accountability or ‘risk work’ therefore offered useful resources to staff to pursue diverse agendas (Horlick-Jones 2005b). In this case Amy, the HCA, became enrolled in the discourse of patient safety and fall prevention in order to negotiate between the moral demand of her patient and calls upon her to complete tasks quickly and move on. As a result, Annie’s sense of worth, identity and fundamentally her dignity is not recognised. The extract below provides a further example of this lack of recognition:

I hear Dorothy saying she would like to go to the toilet and the HCA calls for the SN to come and help her. I hear a disagreement commence – Dorothy says she has a pad and she had definitely ‘gone’. The HCA seems to be disputing this saying ‘there’s nothing there’ and ‘Hold on to the frame’. The patient says again ‘NO I’ve just gone again look’, I’m doing it see. See what I mean’ you see I can feel it, you can’t.’ The HCA then says ‘I’m more concerned with you holding on to the frame.’ The nurse has come out to look for wet wipes and the HCA then tells Dorothy to sit down while she goes to look for the nurse. They return together and both enter the curtains from opposite ends. They tell her they are going to put her on to the bed and to reach back to hold it. I hear Dorothy saying ‘I’m sorry about that’. I hear no response from the staff (Observation: Site 1).

This example is particularly poignant in illustrating the compromises to older people’s dignity that staff make to ensure that specific, measurable risks are managed. Dorothy is extremely concerned about maintaining her self-respect by avoiding the situation she finds herself in. Her plea to use the toilet is ignored by the staff, partly due to their attention being focused on her holding on to the frame. The preservation of her bodily boundaries is breached thus compromising her identity and challenging her dignity.

These examples highlight the implications of system level risk management for day to day care practices that occur on the ward. However, the relationship between the rationalisation of care delivery and the care experience on acute wards must also be seen in the context of broader structural concerns. The number of dependent older patients with co-morbidities who occupy acute hospital beds has risen considerably over the last ten years while nursing levels have stayed much the same. This was the view of many ward staff who described the difficulties experienced in managing work-loads with current staffing levels and the implications of this on the time available to provide the best possible care:

Interviewer: Yeah. Is there anything that stops you doing that? Are there any times when you’re not able to do what you would like to do really?

Staff nurse Jones: It’s time constraints really. I mean you could … Sometimes you know a patient is laid in a wet bed and that you don’t actually have the time to go and clear them up and that’s awful because …

Interviewer: That’s terrible, yes.

Staff nurse Jones: They’re looking at you and it’s obviously uncomfortable and obviously not nice but …

(Ward Staff Interview: Site 2)

Frontline staff provide care in the context of multiple and competing sets of interests, priorities and responsibilities. As Amy’s treatment of Annie illustrates, the regulation of risk is not a straight forward determinant for the quality of care. Systems of governance instead create a hospital ward culture in which it is possible for there to be a distancing between staff and patients, even during moments of intimate care. The distancing of relationships between staff and patients is produced not solely by practices of regulation and risk management; it is produced through the interaction of competing interests and the ways in which staff negotiate them.

Below is an example in which Emma, an HCA, draws on discourses of risk and safety to pursue her decision to deny John his glasses:

John: Can I have a nail brush?

Emma: Yes I’m doing your hands now. We haven’t got a nail-brush but we’ve got these wipes. Oh, your nails need cutting. Oh, here we go okay?

John: What you doing?

Emma: It’s okay, your nails are thick so they’re bending a bit but you’re okay.

John: Oh, stop it.

Emma: You’re okay; they’re just thick I promise. See, that’s it now.

John: They’re bleeding.

Emma: No, they’re fine look. Okay?

John: Where are my spectacles?

Emma: On the side nice and safe.

John: Where?

Emma: Here. (she picks them up and shows them to him)

John: Can I put them on?

Emma: If you put them on, you might roll on to them and hurt your face in the night. Okay?

(Observation: Site 2)

Here, the decision Emma makes in not allowing John to wear his glasses reflects both the organisation of work on acute wards where orientation to the task, timeliness and efficiency prevail and the pre-eminence of patient safety as a risk regime. Emma’s actions are shaped not by a rational calculative decision based only on patient safety performance indicators but instead on an overlapping of local ward micro-politics and the governance of risk (Horlick-Jones 2005a, Zinn 2008). The impact of this decision on John’s sense of dignity is that he is patronised and treated like a child, showing him the glasses but not allowing him to wear them.

The extracts above show how staff’s actions in providing care to acute patients are shaped by institutional, local and personal concerns and that these are carefully negotiated in processes of decision-making. Recognising such connections, the next section highlights the emergence of an institutional concern over accountability in the everyday care practices that occur on acute wards.

## Promoting defensive practice

It was apparent in observations and interviews with staff that, from their perspective, their responsibility to the organisation was to ensure they kept a record of the work undertaken and that this responsibility was as important, if not more important, than the delivery of care itself. A common phrase among staff was ‘if it isn’t written down, it hasn’t been done’.

Although this fear over accountability was present among all staff (even managers and consultants talked about the influence of fears about complaints and litigation in their daily work), the extent of this culture of self-protection differed according to the hierarchies and social positions of various professional groups. For example, the threat of accusation and blame were felt more intently among the nurses and HCAs who felt less able to exercise professional autonomy. This was particularly true for the HCAs who were of relatively low status and therefore more likely to seek the security of self-surveillance through standard protocols and record keeping.

One consequence of this culture of defensiveness, particularly among those providing everyday intimate care to patients, was that staff came to view patients as an enemy to defend themselves against. This has important implications for how ward staff interact with and care for patients:

Two members of staff I hadn’t seen before come up to the nurses’ station and ask Jim (a SN) about Fred, a man on the ward who’d had a fall. He tells them that Fred said he’d fallen ‘but he was back in bed and given how much he struggles in and out of bed I don’t know how he would’ve got himself back into bed’. They nodded and headed down to the bay. Jackie (an HCA) tells me, ‘Fred says he had a fall out of the bed onto the floor right but he never could’ve got off the floor if that’d happened’.

Jim: He did say this morning that he almost fell.

Jackie: That’s why no-one will see to him on their own now.

Jim: It’s turned into a big game of them and us.

(Observation: Site 2)

This defensive practice can result in creating and sustaining a disconnection between staff and patients, challenging the provision of dignified care. The observation below describes another incident involving Fred:

Jackie goes to do some observations on the bay and Fred is really pulling at his incontinence pad, catheter and pyjama bottoms. She goes back and asks Amy to come and help her and they pull the curtains round. I then hear her shout ‘No, you’ve got to keep them on’ followed by ‘No, no no!’

She comes out from behind the curtain and throws it back behind her in anger. As she goes by me she says ‘why do they insist on digging’ (earlier Jackie and Amy talked about patients they called ‘gardeners’ referring to those who go ‘digging’ inside their incontinence pads).

I hear her tell her colleagues that ‘he’s put his hands in his pad and it’s all over his sheets’. She has more sheets and a pad in her hand and as she goes by me again she looks at me and says ‘I don’t know what’s wrong with these people. They start out in this world like babies and end up like babies’.

(Observation: Site 2)

The relationship between Jackie, the HCA, and Fred is one of opposition, ‘them and us’ as Jim described in the previous extract. The construction of Fred as an ‘Other’ is sustained through the institutional culture of blame that permeates ward activities; a culture in which risks automatically hold within them a fault to which blame must be attributed (Douglas 1992). Such a culture creates a ward environment in which staff feel under constant threat. Jackie’s anger in response to this man’s pain, confusion and discomfort is therefore normalised as a natural defence against the potential threat he poses to her.

The ways staff responded to incidents on the wards reflected their fear of being held to account. Patients, as a consequence of this blame culture, become an embodied representation of staff’s potential culpability. Below is an extract taken from a relative’s interview that highlights this fear and its implications for everyday practices of care. It describes a relative’s attempt to raise concerns about her mother’s care:

I mean, there was one time I tried to speak to one of them and she said, ‘Yes?’ and leant against the wall and looked at me and I said, ‘I’m sorry, I’m not going to speak to anybody like that, leaning against the wall and so on, I’d prefer to go somewhere private, can we go to …’ and she said – she said – I don’t know what she thought I was going to say, she said, ‘Well, yes, I suppose so, but I’ll have to have a witness’. And she went off and got someone else and fair enough and we’re sitting, you know, like this, now I don’t know whether she thought I was going to hit her, verbally abuse her or what, but I think, I suppose I felt they were on the defensive which is a shame (Relative Interview: Site 4).

The significance of this approach to the handling of problems on acute wards is not to re-iterate the limitations of bureaucratic systems of litigation and complaints that have been given extensive attention elsewhere (Allsop and Mulcahy 1995, Waring 2005); it is instead to highlight the implications such processes have on ward culture and the threat this poses to the maintenance of patients’ dignity.

## Discussion

The management of risk in healthcare systems has fallen victim to a wider societal trend to attempt to eradicate uncertainties through reasoned calculation. Risk governance in acute trusts is thus part of a wider movement of healthcare rationalisation. Sociological analysis has provided an analytical gaze over such trends to highlight the challenges they pose to social life and people’s everyday relationships and experiences. This article contributes to these analyses by highlighting the experiences of older people on acute hospital wards and illustrates how systems of governance mediate the actions and interactions of staff and patients with de-humanising effects.

The article suggests that providing care with dignity to older people is particularly challenging in the context of risk governance and the wider bureaucratic systems of accountability that permeate the NHS. The current UK government, a coalition government between the Conservative party and the Liberal Democrats has, at least rhetorically, made a commitment to reduce bureaucracy in the NHS (DoH 2010a). However, the proposed acceleration of internal markets and competition are likely to result in economically defined measures of quality and risk, the effects of which are yet to be experienced.

This work shows how systems of risk governance form part of an interactional matrix (Horlick-Jones 2005a) which informs staff’s decision-making in their everyday work. As well as moulding the care practices of staff, risk regimes also make available discursive tools that enable staff to pursue actions that perform to different, competing sets of interests such as the constraints of limited resources, the cohesion of staff groups or existing organisational hierarchies. Staff’s interaction with risk rationalities, as part of this wider interactional matrix are shown to challenge the provision of care with dignity in four ways: distorting practice; disconnecting clinical relationships; promoting defensive practice; and de-humanising patients.

The unintended consequences of rational systems for risk management described here reflect the fundamental paradox of rationalisation as Weber describes it: the more attempts are made to ensure human experiences make sense according to rational precepts, the more likely we are to be faced with the essential irrationality of social life and our experiences of it (Wilkinson 2010). The examples of older people’s care presented show how systems of governance that attempt to bring aspects of care quality under rational control actually distort practice. The systematic calculation of risks in caring work, such as the prevention of falls or the control of infections, are shown to mediate caring relationships and shape practice in unintended ways. The result of this distortion is to deny aspects of older people’s dignity through taking away essential components that help maintain their identity such as control over belongings and personal space and removing possibilities for meaningful social interaction.

Central to the concerns highlighted in this work are the ways in which risks – faced by older users of acute health services – are being ‘operationalised’. Power (2004) describes the ways in which societal risks, such as accidental harms done to patients in the course of clinical treatment or care, are being increasingly overridden by ‘secondary’ or ‘systems risk’. Such ‘secondary’ risks include litigation, failure to meet targets and accompanying punitive financial repercussions or reputational risk to the acute trust. This article shows that through the enrolment of staff in discourses of institutional risk, the opportunities for undignified care are exacerbated. Older people’s identity and subsequently their dignity are obscured by staff’s negotiation of governance strategies and the care practices that ensue.

Risk governance systems and their negotiation in everyday practice can distance staff from the essential irrationality of caring relationships and instead attempt to instil expectations of control and manageability. The repercussions of this for staff are that they are less able to respond to the inherent uncertainties and irrationalities of providing care. Just as Castel (1991) suggests, the role of the practitioner in direct response to their patient is reduced. Through both their subjection to and enrolment in the ‘risk techniques’ (Dean 1999) of acute trusts- including measurement, audit, targets and practices of accountability- staff actions are shaped less by the presence of their patients as full persons (Cresswell 1996) or moral demands (Bauman 1990) and are instead shaped by their negotiations with the institutional concerns of accountability and performance.

The tendency among staff of self-protection and defensiveness, highlighted by Jackie’s response to John, her patient who goes ‘digging’, reflects the broader systemic approach across acute trusts of managing ‘secondary risks’ of blame, reputation and punitive financial sanctions to the organisation. This institutional culture of blame is mediated through local ward cultures so that staff who provide the most intimate care to older people feel under most threat. The positioning of patients as an enemy who the staff must protect themselves against is one of the most significant repercussions of a culture in which the management of ‘secondary risks’ (Power 1997) manifest themselves in everyday care provision.

Most significantly, the mediation of risk regimes into relations of care between staff and patients can result in situations where older people’s dignity is compromised. The examples of indignity, in which Dorothy and Annie are denied control of their own bodies, highlights the importance of sociological understandings of risk for understanding how the organisation of health services can produce cultures of care that lack humanity.

This article highlights how risk regimes reduce possibilities for meaningful caring relationships to develop, relationships that maintain patients’ positive personal and social identities (Goffman 1963). The mediation of risk rationalities into the micro-politics of acute wards and staff decisions about care delivery are shown to detract from older people’s visibility as people with a past and a future, diminishing their sense of self-worth and threatening their dignity.

Techniques and strategies for risk management in healthcare are shown not to act as direct control for professionals to resist, but indirect regulation (Rose and Miler 1992, Shore and Wright 1999, Flynn 2002). As this article has demonstrated, whether staff subvert or adhere to forms of governance and regulation, or indeed perform ‘risk work’ to pursue their own agendas, risk regimes shape the conditions of possibility for acute care provision. These conditions are shown to reduce the possibilities for meaningful caring relationships between practitioners and patients in which the dignity of older people is maintained and instead promotes practices that maintain the system rather than meeting the needs of the people the system purports to serve.

This article situates the problem of undignified care for older people within a social context and challenges explanations that identify the moral failings of individuals. The examples presented illustrate how risk techniques, as part of a wider movement of healthcare rationalisation, reduce the possibilities for meaningful interactions between staff and patients that support identity work and maintain dignity. In particular, the mediation of institutional risk and accountability into everyday caring relationships not only reduce patients as a moral demand (Bauman 1990) but can even constitute patients as an enemy, posing a potential threat to those who care for them. In broadening the scope of the debate in understanding the problem of undignified care, this article re-asserts the importance of a sociological imagination (Wright-Mills 1959) to re-connect the social and political with the personal.
